# Trial of Oxygen Delivery on Cardiopulmonary Bypass and Major Clinical Outcomes

**DOI:** 10.1016/j.atssr.2024.05.012

**Published:** 2024-06-07

**Authors:** Rawn Salenger, Clifford E. Fonner, Christa Kampert, Amanda Rea, Charles Evans, Rakesh C. Arora

**Affiliations:** 1 Department of Surgery, University of Maryland School of Medicine, Towson, Maryland; 2 Division of Cardiac Surgery, University of Maryland St. Joseph Medical Center, Towson, Marlyand; 3 Maryland Cardiac Surgery Quality Initiative, Baltimore, Maryland; 4 Harrington Heart and Vascular Institute, University Hospital – Cleveland/Case Western Reserve University, Cleveland, Ohio

## Abstract

**Background:**

Low oxygen delivery (DO2) on cardiopulmonary bypass has been associated with acute kidney injury. We sought to determine the association of intraoperative DO2, postoperative length of stay, and major postoperative events.

**Methods:**

DO2 values were calculated in 845 patients after initiation, and every 30 minutes on bypass. Pump flows were increased for DO2 < 280 mL O2/min/m^2^, but care was not otherwise adjusted. Patients were retrospectively separated into 3 groups based on DO2 values: Group A, all readings ≥280 mL O2/min/m^2^; Group B, ≥1 reading <280 mL O2/min/m^2^; Group C, ≥2 readings <280 mL O2/min/m^2^. Patient outcomes were analyzed.

**Results:**

We analyzed 845 consecutive adult cardiac cases. Group B patients had a higher Society of Thoracic Surgeons Predicted Risk of Mortality compared with Group A (1.9% vs 1.2%, *P* < .001), and this effect was amplified for Group C patients (2.2%, *P* < .001). Postoperative length of stay was lowest for Group A patients (5.2 days) compared with Group B (6.6 days, *P* < .001) and Group C (7.0 days, *P* < .001). Overall complications rates were low, although Group A patients experienced lower rates of prolonged ventilation (3.5%) compared with Group B (6.5%, *P* = .04) and Group C (9.2%, *P* = .004). Multivariable regression analysis confirmed that DO2 above threshold was associated with significantly reduced rates of prolonged ventilation and postoperative length of stay. Other outcomes were similar between groups.

**Conclusions:**

Even a single DO2 value below threshold was associated with excess prolonged ventilation and postoperative length of stay, but not other outcomes.

**In Short**

▪ Goal-directed perfusion establishes a threshold oxygen delivery for cardiopulmonary bypass.
▪ Maintaining oxygen delivery ≥280 mL O_2_/min/m^2^ may correlate with faster recovery.

For the patient undergoing cardiac surgery, goal-directed perfusion refers to performing cardiopulmonary bypass (CPB) guided by maintaining a minimum oxygen delivery (DO2) threshold in order to optimize end-organ perfusion and potentially reduce acute kidney injury (AKI).^1^, ^2^, ^3^, ^4^, ^5^ The critical threshold for DO2 on CPB has been demonstrated to be in the range of 225 to 272 mL/min/m^2^, and investigators have targeted a DO2 threshold >280-300 mL/min/m^2^.^1^^,^^3^, ^4^, ^5^, ^6^, ^7^ DO2 below this threshold has been associated with postoperative AKI, primarily stage 1. The impact of goal-directed perfusion on other major postoperative events is uncertain. The goal of this study was to examine the association of DO2 on CPB with major Society of Thoracic Surgeons (STS) postoperative events and postoperative length of stay (pLOS) in adult cardiac surgery patients.

## Patients and Methods

### Patient Selection, Outcomes, Protocol

Requirement for individual patient consent was waived by the institutional review board. Data were collected prospectively from January 2021 to March 2023 for adult, on-pump isolated coronary or valve surgery. DO2 values were calculated after initiation, and every 30 minutes while on CPB. Patients undergoing combined valve/coronary surgery, hypothermic circulatory arrest, or extracorporeal membrane oxygenation were excluded. CPB was conducted according to institutional protocols, keeping the cardiac index between 2.2 and 2.4 L/min, mean arterial pressure >65 mm Hg, and temperature 32°C. Cases were performed on pump with cardioplegic arrest. If DO2 values fell below 280 mL O2/min/m^2^, pump flow was increased, but transfusion of packed red blood cells was not utilized to increase DO2. Patients were retrospectively separated into 3 groups based on whether DO2 fell below the threshold of 280 mL O /min/m^2^: Group A, all readings ≥280 mL O2/min/m^2^; Group B, ≥1 reading <280 mL O2/min/m^2^; Group C, ≥2 readings <280 mL O2/min/m^2^. Patient outcomes were analyzed regarding renal failure, stroke, re-exploration for bleeding, deep sternal wound infection, prolonged ventilation, operative mortality, and pLOS. All postoperative events were defined according to STS Adult Cardiac Surgery Database definitions. After identifying significant associations with outcomes using observational data, we performed multivariable regression analyses to identify outcomes independently associated with DO2.

### Statistical Analysis

Baseline characteristics and outcomes were compared between groups. The Student *t* test was used for normally distributed variables, the Wilcoxon rank sum test was used for nonnormally distributed variables, and the χ^2^ test of independence was used for categorical variables. Multivariable linear regression was utilized to analyze the associations between multiple predictor variables and continuous outcomes, and multivariable logistic regression was performed for categorical outcome metrics. *P* value ≤ .05 was considered significant.

## Results

A total 845 consecutive adult coronary bypass and valve cases were analyzed (Supplemental Table 1). Group A patients were younger, with fewer comorbidities, and a lower overall STS Predicted Risk of Mortality score compared with Group B patients (1.2% vs 1.9%, *P* < .001), and this effect was amplified for Group C (1.2% vs 3.1%, *P* < .001) (Table 1). Mean preoperative creatinine was lowest and preoperative hemoglobin was highest for Group A patients (Table 1). Mean DO2 values were higher at all time points in Group A (range, 328-332.8 mL O2/min/m^2^) compared with Group B (range, 252.6-266.3 mL O2/min/m^2^; *P* < .001) and Group C (range, 240.1-253.7 mL O2/min/m^2^; *P* < .001) (Supplemental Table 2).

**Table 1:** Patient and Operative Characteristics

Characteristic	Group A	Group B	*P* Value (A vs B)	Group C	*P* Value (A vs C)
Mean age, y	64.8	68.5	<.001	68.7	<.001
Female sex	15.3	46.0	<.001	53.9	<.001
White race	86.2	78.0	.002	74.3	<.001
Black race	10.1	16.5	.006	19.7	.001
Hispanic ethnicity	1.5	2.0	.62	2.7	.33
Mean BMI	30.4	28.9	<.001	28.9	.005
Hypertension	86.9	89.0	.38	88.2	.69
Cerebrovascular disease	23.7	32.0	.008	34.9	.009
Diabetes	44.4	47.9	.33	52.6	.07
COPD	20.7	23.9	27	27.0	.10
Preoperative Hemoglobin, mean	13.9	12.1	<.001	11.7	<.001
Creatinine, mean	1.00	1.32	<.001	1.30	<.001
Ejection fraction, mean	53.9	54.7	.29	54.0	.94
STS Predicted Risk of Mortality score	1.2	1.9	<.001	3.1	<.001
Isolated CABG, % (n)	89 (479)	90 (279)	.67	91 (138)	.61

Values are presented as %, unless otherwise noted.

BMI, body mass index; CABG, coronary artery bypass grafting; COPD, chronic obstructive pulmonary disease; Group A, all DO2 readings ≥280 mL O2/min/m^2^; Group B, ≥1 reading <280 mL O2/min/m^2^; Group C, ≥2 readings <280 mL O2/min/m^2^; STS, The Society of Thoracic Surgeons.

### Exploratory Analysis of Unadjusted Data

Group A patients experienced lower rates of prolonged ventilation (3.2%) compared with Group B (7.4%, *P* = .005) and Group C (9.2%, *P* = .002). Observed rates of renal failure, stroke, mediastinitis, and operative mortality were similar for all groups (Figure). Postoperative LOS was shorter greater for Group A patients vs Group B (5.2 days vs 6.6 days, *P* < .001), with Group C demonstrating the longest ppLOS (7.0 days, *P* < .001). Any AKI, as defined by the Kidney Disease Improving Global Outcomes criteria, was significantly different between Group A and Group B and Group C in unadjusted data. Rates of AKI were 12.1% for Group A vs 23.3% for Group B (*P* < .001) and 27.0% for Group C (*P* < .001). The incidence of acute renal failure, stroke, reexploration for bleeding, deep sternal wound infection, and operative mortality were similar between groups (Table 2).

**Figure:**
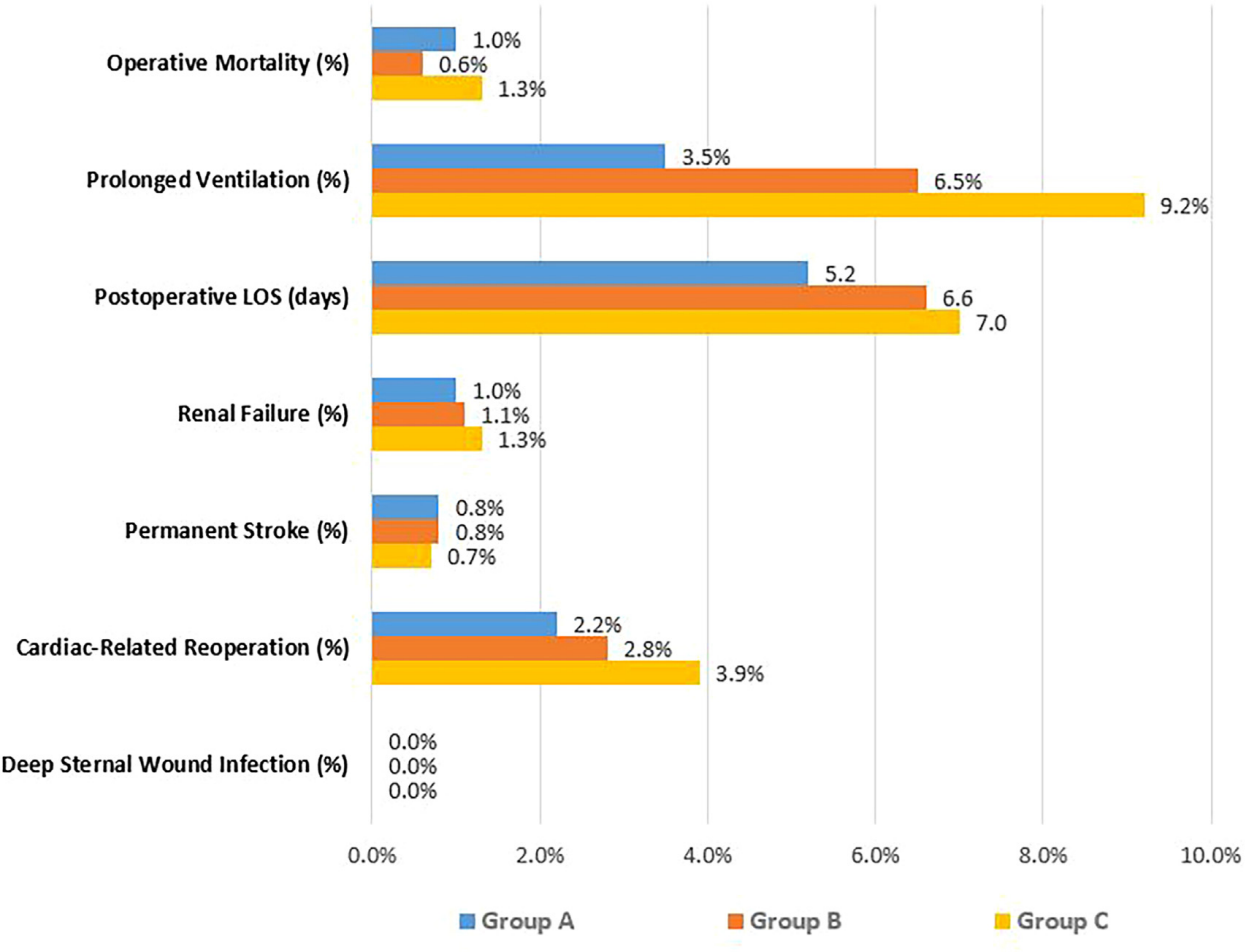
Postoperative length of stay (LOS) and Society of Thoracic Surgeons outcomes by oxygen delivery (DO2) group. Prolonged ventilation significantly increased in Groups B and C compared with Group A. (Group A, all DO2 readings ≥280 mL O2/min/m^2^; Group B, ≥1 reading <280 mL O2/min/m^2^; Group C, ≥2 readings <280 mL O2/min/m^2^.)

**Table 2:** The Society of Thoracic Surgeons Outcomes and Postoperative Length of Stay for Groups A, B, and C

Outcome	Group A	Group B	*P* Value (A vs B)	Group C	*P* Value (A vs C)
Atrial fibrillation	34.3	32.7	.63	36.2	.67
Prolonged ventilation	3.2	7.4	.005	9.2	.002
Renal failure	0.9	1.3	.62	1.3	.68
Permanent stroke	0.7	1.0	.73	0.7	.91
Reoperation	2.1	3.2	.29	3.9	.18
Deep sternal infection	0.0	0.0	…	0.0	…
Failure to rescue	0.9	0.3	.31	0.7	.75
Operative mortality	0.9	0.6	.66	1.3	.68
Mean postoperative length of stay, d	5.2	6.6	<.001	7.0	<.001
Acute kidney injury	12.1	23.3	<.001	27.0	<.001

Values are presented as %, unless otherwise noted.

Group A, all DO2 readings ≥280 mL O2/min/m^2^; Group B, ≥1 reading <280 mL O2/min/m^2^; Group C, ≥2 readings <280 mL O2/min/m^2^.

### Multivariable Regression Analysis

The association of DO2 falling below 280 mL/min/m^2^ with prolonged ventilation, pLOS, and AKI was studied by creating 3 separate multivariable regression models with similar variables utilized for each model was subsequently examined (Supplemental Table 3). Multivariable regression analysis demonstrated that the number of DO2 readings <280 mL/min/m^2^ during CPB was independently associated with prolonged ventilation (*P* = .04) and longer pLOS (*P* < .02). Other variables independently associated with prolonged ventilation included chronic obstructive lung disease, preoperative ejection fraction, elective case status, and CPB time. Postoperative LOS was also found to be independently associated with the variables increasing age, preoperative creatinine, chronic obstructive pulmonary disease, preoperative ejection fraction, and CPB time. The association between threshold DO2 and the incidence of AKI did not reach significance in the multivariable model with a *P* value = .06. Variables found to be independently associated with developing AKI included increasing age, female sex, increasing preoperative creatinine, diabetes, chronic obstructive pulmonary disease, preoperative anemia, and CPB time (Supplemental Table 3).

## Comment

This is one of the largest trials examining DO2 during CPB and major postoperative outcomes after cardiac surgery. Maintaining DO2 ≥ 280 mL/min/m^2^ was associated with a reduced incidence of prolonged ventilation, shorter pLOS, and trended toward reduced AKI. Prior randomized and observational trials have established an association between CPB DO2 and stage 1 AKI,^2^^,^^3^^,^^5^^,^^8^ and possibly more advanced AKI.^1^^,^^4^ In our study, DO2 below threshold appeared close to significant as an independent association with AKI (*P* = .06). The reason why AKI, contrary to prior studies, failed to reach significance is unclear, but possibly this study was underpowered. Despite including 845 patients, the overall incidence of AKI was relatively low, at 16%. Another possible explanation may be that pump flows were aggressively increased to correct DO2 if values fell below 280 mL/min/m^2^. Although transfusion of packed red blood cells will increase DO2, transfusion has been shown to disproportionately increase the risk of AKI relative to anemia.^2^^,^^8^^,^^9^ Furthermore, our cohort was 90% patients undergoing coronary artery bypass grafting surgery, and the effect of DO2 may be more profound for patients undergoing valve or combined coronary and valve surgery.^6^ Additional study is needed to better elucidate the exact relationship between DO2 and cardiac surgery-associated AKI.

The data from this analysis reflect that the incidence of DO2 falling below the threshold value of 280 mL/min/m^2^ during CPB was inversely proportional to STS Predicted Risk of Mortality risk score. Low DO2, therefore, was a surrogate for higher-risk patients, and we utilized multivariable regression to demonstrate an independent effect of DO2 on prolonged ventilation and pLOS. Importantly, prior studies suggest that maintaining threshold DO2 in patients with elevated baseline glomerular filtration rate or preoperative anemia may improve outcomes.^1^^,^^6^ The current trial adds to current literature by demonstrating an independent association between maintaining DO2 during CPB above a threshold of 280 mL/min/m^2^ and the major STS postoperative event of prolonged ventilation as well as pLOS in the multivariable models. These important findings increase the potential significance of maintaining CPB DO2 above a critical threshold. A prior study also showed a correlation with DO2 below a threshold of 280 mL/min/m^2^ and prolonged ventilation, but did not examine pLOS.^10^ Of the outcomes studied, we are not able to explain why prolonged ventilation and pLOS were the 2 metrics most associated with low DO2 during CPB. One potential reason could be that suboptimal end organ perfusion during CPB leads to greater organ injury and inflammatory response, requiring a generally longer recovery period.

Our study has the limitations imposed by any retrospective design. Although we performed multivariable regression, we cannot account for unmeasured risk factors or unrecognized bias. In addition, our sample size was insufficient to illustrate a difference in some of the less-common STS risk-adjusted postoperative events. Additionally, the amount of time spent below threshold DO2 may be important regarding postoperative outcomes and these data were unavailable. However, we were still able to demonstrate an independent association between lower DO2 values and both prolonged ventilation and pLOS. Our findings, in conjunction with results of prior studies on goal-directed perfusion, merit further investigation in a randomized trial.

In conclusion, in this study, an independent association between DO2 <280 mL O2/min/m^2^ on CPB, prolonged ventilation, and pLOS was observed in adult patients undergoing coronary artery bypass grafting and valve surgery. Additional study is required to identify the optimal intraoperative DO2 level and clinical implications.
