# Supplementary material for: Trial of Oxygen Delivery on Cardiopulmonary Bypass and Major Clinical Outcomes
Source: Ann Thorac Surg Short Rep. 2024 Jun 7;2(4):855–9. doi: 10.1016/j.atssr.2024.05.012 (PMC11708650; doi:10.1016/j.atssr.2024.05.012)
Supplement: Supplementary Table 1-3 [file mmc1.docx]

**DO2 Supplemental Material: Appendix A-C**

**Supplemental Table 1:** Volume by Procedure Type and Number of DO2 Readings Under 280

|  | **N** | **DO2 Groups** | | |
| --- | --- | --- | --- | --- |
|  |  | **Group A** | **Group B** | **Group C** |
| **Isolated CABG** | 758 | 479 | 279 | 138 |
| **AV Replacement** | 40 | 29 | 11 | 4 |
| **MV Repair** | 36 | 22 | 14 | 8 |
| **MV Replacement** | 11 | 6 | 5 | 2 |
| **Total** | **845** | **536** | **309** | **152** |

**Legend:** CAB = coronary artery bypass grafting; AV = aortic valve; MV = mitral valve. **Group A:** All DO2 calculations ≥ 280 ml/min/m^2^, **Group B:** ≥ 1 calculation under 280 ml/min/m^2^, **Group C:** ≥ 2 calculations under 280 ml/min/m^2^.

**Supplemental Table 2:** DO2 data compared between cohorts

|  | **Group A** | **Group B** | **p value**  **(A vs. B)** | **Group C** | **p value**  **(A vs. C)** |
| --- | --- | --- | --- | --- | --- |
| Mean DO2 #1 | 329.6 | 266.3 | < 0.001 | 253.7 | < 0.001 |
| Mean DO2 #2 | 328.0 | 252.6 | < 0.001 | 240.1 | < 0.001 |
| Mean DO2 #3 | 332.8 | 263.3 | < 0.001 | 246.7 | < 0.001 |

**Group A:** All DO2 calculations ≥ 280 ml/min/m^2^, **Group B:** ≥ 1 calculation under 280 ml/min/m^2^, **Group C:** ≥ 2 calculations under 280 ml/min/m^2^. All DO2 values displayed as ml/min/m^2^.

**Supplemental Table 3:** Multivariate regression results

|  | **Prolonged Ventilation** | | **Postoperative LOS** | | **AKI** | |
| --- | --- | --- | --- | --- | --- | --- |
|  | **Coefficient** | **p value** | **Coefficient** | **p value** | **Coefficient** | **p value** |
| **# DO2 Readings < 280** | 0.023 | **0.04** | 0.565 | **0.02** | 0.035 | 0.06 |
| **Patient Age** | 0.001 | 0.32 | 0.061 | **< 0.001** | 0.005 | **< 0.001** |
| **Female Gender** | 0.020 | 0.25 | 0.637 | 0.11 | -0.085 | **0.005** |
| **Preop Creatinine** | 0.014 | 0.05 | 0.684 | **< 0.001** | 0.064 | **< 0.001** |
| **Diabetes** | 0.000 | > 0.99 | 0.412 | 0.22 | 0.086 | **0.001** |
| **Hypertension** | 0.032 | 0.16 | 0.641 | 0.20 | 0.072 | 0.06 |
| **COPD** | 0.043 | **0.01** | 1.190 | **0.003** | 0.064 | **0.03** |
| **CVD** | 0.025 | 0.14 | 0.701 | 0.06 | -0.005 | 0.87 |
| **Preop Hematocrit** | 0.002 | 0.21 | 0.035 | 0.20 | -0.009 | **< 0.001** |
| **EF** | -0.003 | **< 0.001** | -0.064 | **< 0.001** | -0.001 | 0.38 |
| **Previous CABG** | -0.069 | 0.57 | -1.699 | 0.53 | -0.198 | 0.33 |
| **Previous MI** | -0.013 | 0.40 | 0.205 | 0.57 | -0.016 | 0.56 |
| **Elective Status** | -0.037 | **0.02** | -0.365 | 0.33 | 0.006 | 0.83 |
| **CPB Time** | 0.000 | **0.02** | 0.013 | **0.01** | 0.001 | **0.01** |
